# Inhibitive effect of *Urginea epigea* methanolic extract and silver/zinc oxide nanoparticles on *Aspergillus* and aflatoxin production

**DOI:** 10.1371/journal.pone.0320454

**Published:** 2025-04-24

**Authors:** Martha Cebile Jobe, Mulunda Mwanza

**Affiliations:** 1 Department of Animal Health, North-West University, Mahikeng Campus, Mmabatho, Mahikeng, South Africa; 2 Food Security and Safety Focus Area, North-West University, Mahikeng Campus, Mmabatho, Mahikeng, South Africa; Annamalai University, INDIA

## Abstract

Food crops contaminated with *Aspergillus flavus* due to aflatoxins can be hazardous for both humans and animals, hence endeavours are being explored to find natural antifungals to combat the contamination and mycotoxin issue. The current study used the agar dilution method to assess the effect of *Urginea epigea* methanolic extract and biosynthesized silver-zinc oxide nanoparticles on the toxigenic strain of *Aspergillus*. In the experiment, an aflatoxin-producing strain was used, and potato dextrose agar was diluted with methanolic extract from *U. epigea* and silver/zinc oxide nanoparticles at concentrations of 0, 6.5, 12, 25, and 50 mg/mL, respectively. Mycelia growth diameters were measured to test inhibitory activity. A significant decrease in fungal growth was observed at different concentrations (*P* < 0,05) when compared to the control. At 50 mg/mL, the extract of *U. epigea* significantly reduced the growth of *A. flavus* by 100%. PCR data shows that the expression of *aflD* and *aflR* was significantly downregulated (*P* < 0.005) by the treatments, with *U. epigea* having a 50fold decrease when compared to Ag/ZnO nanoparticles. Compared to the controls, Ag/ZnO nanoparticles down-regulated the expression of *aflD* and *aflR* in *A. flavus* by more than 30-fold. However, there was less expression by nanoparticles, as evidenced by the sequence alignment. *A. flavus* growth and aflatoxin B_1_ production were both considerably suppressed by *U. epigea* methanolic extract, through the presence of phytochemicals thus has the potential to be employed as an alternative antifungal agent to control aflatoxigenic fungus. The study recommends investigating and extracting the active compound present in the *U. epigea* bulb.

## 1. Introduction

Feed contaminated with toxigenic fungi has shown to be a formidable opponent in the fight against negative impacts on animal and human health. As a result, regulatory entities like the Food and Agriculture Organization and the World Health Organization have put in place measures to limit fungi production [1,2]. Fungi are filamentous organisms that contaminate agricultural products, and some have the potential to produce secondary metabolites known as mycotoxins. Mycotoxins are harmful to humans and animals, and they have a significant economic impact [3]. *Aspergillus flavus* and *Aspergillus parasiticus* are significant food and animal contaminants as they produce mycotoxins known as aflatoxins which are considered the most carcinogenic, mutagenic, and teratogenic secondary metabolites [4]. When cultured on agar, *Aspergillus* species produce a vivid yellow-green conidial hue, which allows them to be easily differentiated morphologically. These fungi invade and grow in commodities like peanuts, corn, and cottonseed, the resulting contamination with aflatoxins often renders the commodities unfit for consumption [5]. *A. flavus* isolates have been found to produce aflatoxins at varying levels, enhancing their toxigenic potential. These compounds cause liver and kidney damage (hepatotoxic and nephrotoxic) in humans and most experimental animal species studied, suppressing immunological function (immunosuppression), leading to human poisoning and, in some cases, death [6].

Many consumers prefer food that is free of preservatives and identify healthy and safe food with products that are fresh or barely processed [7]. Since the general population is becoming more aware of the polluting, lingering, carcinogenic, and phytotoxic consequences of many synthetic fungicides, the use of alternative indigenous agents to manage phytopathogenic fungi is gaining popularity [8]. Removal of mycotoxins from contaminants emolument is a critical aspect of nutrition research and a variety of physical, chemical, and biological aspects have been used and somewhat successful [9]. It has been reported that phytochemicals have inhibitory effects on most of the microorganisms *in vitro* [10]. Much effort has been expended in the quest for antifungal compounds derived from natural sources for use in food and grain, and several naturally occurring antimicrobials in plants have been identified [11,12]. Several research has been conducted on the antimicrobial effects of medicinal plants, and they are reported to be good antimicrobial agents. Thus, this study devoted to use *Urginea epigea* bulb extract, which is widely distributed in Southern Africa and exhibits extraordinary drought tolerance. Bulbs are generally present above the soil surface, as suggested by the specific epithet, in contrast to its nearest relative *Drimia altissima* (syn. *Urginea altissima*), which has mostly subterranean perennating organs. The bulb of *U. epigea*, on the other hand, generally causes skin irritation when handled, indicating the presence of bufadienolides [13]. *Urginea epigea* is known for its antibacterial and antioxidant activities. Cardiotonic and anti-cancer qualities are their most studied pharmacological actions [14,15] but they also have blood pressure elevating, anti-angiogenic, anti-viral, immunomodulatory, anti-inflammatory, and anti-bacterial capabilities [14,16]. The presence of phytochemicals in the *U. epigea* bulb has been reported by Jobe *et al.* [17] through the presence of functional bonds using Fourier transform infrared spectroscopy. However, phytochemicals are chemically unstable and easily degradable upon exposure to environmental conditions such as temperature, oxygen, pH, and light before being absorbed [18]. Therefore, it is necessary to protect these bioactive compounds in order to ensure their controlled release at the right place and time. Nanoencapsulation is one of the most effective technologies for entrapping, protecting, and delivering bioactive compounds into biological systems [19–21] and is gaining more attention in biomedical applications. Nanotechnology has versatile benefits for targeted site-specific delivery and efficient absorption through cells. Extensive literature has shown the antimicrobial activities of different nanomaterials such as copper, zinc, magnesium, and gold but silver nanoparticles have proved to be more effective against bacteria, fungi, and viruses [22]. However, it is toxic at high concentrations thus a nanocomposite with zinc oxide was employed in this study because of its eco-friendliness, nontoxicity, and high chemical stability, no research has investigated the antifungal effect of Ag/ZnO nanocomposite on *A. flavus* and aflatoxin production. Hence, the present study aimed to investigate the antifungal activity of *U. epigea* bulb methanolic extract and Ag/ZnO nanoparticles encapsulated with *U. epigea* against the *Aspergillus* strain.

## 2. Materials and methods

### 2.1. Materials

A standardized *Aspergillus flavus* (MG659624) was obtained from the Agricultural Research Council, Pretoria, South Africa. Chemicals, potato dextrose agar (PDA), yeast extract, sucrose, and agar were purchased from Sigma Aldrich. Silver/Zinc Oxide nanoparticles (both biosynthesized and conventional) were synthesized from the Department of Chemistry, Faculty of Physics and Chemistry, North West University by Jobe *et al*. [17].

### 2.2. Collection of *U. epigea*

*Urginea epigea* was harvested from Ermelo, Mpumalanga Province South Africa at a latitude of -26° 31′59.99″ S and longitude of 29° 58′59.99″, kept in brown bags, and sent to the botanist for identification. *U. epigea* bulb was washed under tap water and rinsed with distilled water, before being chopped, and air-dried at room temperature, to avoid volatile compounds from degrading [23]. The study was approved by the Institutional Review Board Ethics Committee of NORTH WEST UNIVERSITY (NWU-00803–21-A5 in March 2021).

### 2.3. Qualitative analysis of the phytochemicals in *U. epigea* bulb

The phytochemical screening of *U. epigea* bulb was determined using Ejekeme *et al.* [24] with minor modifications.

#### 2.3.1. Test for saponins.

A mixture of 0.3 g of powdered *U. epigea* bulb and 30 mL of distilled water was heated in a water bath for 10 min before being filtered through 150 mm Whatman filter paper (Lasec). To make a stable, long-lasting foam, 10 mL of the filtrate was mixed with 5 mL of distilled water and briskly shaken. Before testing for emulsion formation, the froth was vigorously agitated, and three drops of olive oil were added.

#### 2.3.2. Test for flavonoids.

Bulb powder (0.3 g) was weighed into a beaker, combined with 30 mL distilled water, heated for 2 h in a water bath, and filtered through 150 mm Whatman filter paper (Lasec). 5 mL of 1 M ammonia solution was added to 10 mL of each extract’s aqueous filtrate, followed by 5 mL of concentrated tetraoxosulphate (VI) acid. The presence of flavonoids was indicated by a yellow coloration.

#### 2.3.3. Test for terpenoids.

Bulb powder (0.3 g) was weighed into a beaker and mixed with 30 mL distilled water, then set aside for 2 h. To create a layer, approximately 5 mL of the extract was mixed with 2 mL of chloroform and 3 mL of concentrated tetraoxosulphate (VI) acid. A reddish-brown coloration that forms at the interface would indicate the presence of terpenoids.

#### 2.3.4. Test for tannins.

*Urginea epigea* bulb powder (0.3 g) was weighed and then heated in 30 mL of water in a water bath for 10 min before and filtered through 150 mm Whatman filter paper (Lasec). Three drops of 0.1% ferric chloride were added to 5 mL filtrate and the colouration was checked for brownish green or blue-black.

### 2.4. Preparation of *U. epigea* bulb extract

Extraction of *U. epigea* bulb was done following Bhalla *et al.* [25] and Baeshen *et al*. [26] with minor modifications, where bulb powder (5 g) was weighed and placed in a glass beaker with 100 mL of 50% methanol in distilled water, the mixture was placed in an orbital shaker at 150 rpm at room temperature for 24 hr. After 24 hr, it was filtered through 150 mm Whatman filter paper (Lasec). The filtrate was then evaporated to eliminate the solvent using a rotary evaporator under reduced pressure at 64 °C. The residue (crude extract) was dried to consistent weight, yielding a green solid suspension, and kept at 20 °C until use.

### 2.5. Silver/zinc oxide nanoparticles

Silver/Zinc Oxide nanocomposite used in this study was prepared by Jobe *et al.* [17]. Briefly, 20 mL of *U. epigea* bulb extract was utilized as a reducing agent while 0.05 M of zinc acetate and 2% silver nitrate were utilized as metal precursors for the synthesis of nanoparticles. The mixture was placed on a magnetic stirrer for 90 min, followed by centrifugation, rinsing, and drying. Using scanning electron microscopy, transmission electron microscopy, and X-ray diffraction, the dried nanoparticles were found to be spherical with a mean size of 33.5 nm and a crystallite size of 25.15 nm, respectively.

### 2.6. Aflatoxigenicity of the *Aspergillus* strain

#### 2.6.1. Yeast extract agar.

The *A. flavus* isolate was grown on YES agar (2% yeast extract, 15% sucrose, and 1.5% agar) as single colonies in the centre of the solidified agar and incubated at 30^o^C for 7 days. On day 3, the toxigenicity was tested using ammonium solution where 2 mL was added to the plates in an upside-down position while allowing the vapour to react with the toxins in the agar for 10 min, the procedure was repeated on day 7. Colour change into a pink to red colour on the underside of the colony was used to determine the toxicity of the isolate [27].

#### 2.6.2. Coconut agar media.

Coconut agar was prepared by mixing two hundred grams of desiccated coconut and 1.5 g agar followed by autoclaving at 121 °C for 15 min. Single spores of the *Aspergillus* strains were inoculated at the centre of the plates and incubated at 30 °C for 7 days [28]. Aflatoxigenic test was done on day 3 and 7 by putting plates under an ultraviolet light at 365 nm. The colony’s fluorescent ring served as proof that the strain produced aflatoxin.

#### 2.6.3. Thin layer chromatography.

The positive isolates were cultured on YES agar for 7 days at 25 °C. Agar with mycelia were homogenized in a blender, and then 250 mL of a methanol-water (9:1, v/v) solution containing 3 g of sodium chloride was used for extraction [29]. Following vigorous shaking in the presence of 100 ml of hexane,150mm Whatman filter paper (Lasec) was used to filter the slurry. A 25 mL of the filtrate was extracted with 25 mL chloroform. After using anhydrous sodium sulphate to dry the filtrate, it was nearly dried by being evaporated in a steam bath. Chloroform (200 µL) was used to redissolve the residue and 5 µL was spotted on the TLC plates alongside aflatoxin standards. A developing solvent system consisting of acetone and chloroform (1:9, v/v) was used, and the TLC result was visualized under 365 nm UV radiation. Confirmation of aflatoxin identity was then achieved by spraying the plate with 25% sulphuric acid.

### 2.7. Inoculation of *Aspergillus, U. epigea* extract, and Ag/ZnO nanoparticles

*Aspergillus flavus* stored in glycerol at -20 °C was revived in potato dextrose agar (prepared following the manufacturer’s instructions where 39.5 g was dissolved in 1000 mL of sterile water and autoclaved at 120 °C for 15 min) and incubated for 5 days at 30 °C. *Urginea epigea* crude extract and Ag/ZnO nanocomposite were redissolved in methanol: water (50:50/v/v). The agar dilution method was used where PDA plates were amended with various concentrations of *U. epigea* extract, biosynthesized Ag/ZnO, and conventional Ag/ZnO nanoparticle solution (6.5, 12.5, 25, and 50 mg/mL) and allowed to solidify before inoculation. Inoculation was carried out by adding 100 µL (2 x10^-3^ spores/µL) [30] spore suspension of *A. flavus* strain. All plates were incubated in triplicate for each concentration at 30 °C for 7 days. *A. flavus* growth observation was done regularly during the incubation period. Methanol was used as a negative control. On day 7, *A. flavus* growth diameter was measured by measuring all the uneven growth in the plate and taking the average. The mycelial growth inhibition percentage was calculated according to the formula in (Equation 1):


$$Inhibitoryactivity\%=\frac{Dc-Dt}{Dc}x100$$ (1)

Where:

Dc is the mean diameter of the colony in the control (mm)

Dt is the mean diameter of the colony in the treatment (mm).

This experiment was repeated three times under the same conditions and all treatments were performed independently in triplicate.

### 2.8. DNA extraction

Fungal spores were scrapped after 7 days to extract DNA using the Zymo Research Quick-DNA™ Fungal/Bacterial Miniprep Kit (The Epigenetics Company™, United States) in accordance with the manufacturer’s instructions. Briefly, mycelia and spores were treated with 750 µL of lysis solution before having their cells disrupted with a gene disruptor (Scientific Industries Inc., USA). Supernatant from the lysed mixture was filtered and collected after being centrifuged at 10,000 rpm for 10 min. The DNA binding solution was used to separate the DNA from the remaining cell components. The DNA pellet was re-suspended in 100 µL of elution buffer after the supernatant was discarded. The quality of the DNA was determined using 1% agarose gel electrophoresis [31].

### 2.9. Molecular identification of aflatoxin producers

Using the primer pairs and conditions listed in Table 1, the *aflD, aflM, aflP*, and *aflR* genes were amplified by PCR [32–35]. The following volumes were used; 0.5 µL of each 25 mM primer (Inqaba Biotech, South Africa), 12.5 µL of 2x PCR mix (OneTaq®QuickLoad, Biolabs Inc, New England), 5 µL of the extracted DNA, and enough nuclease-free water to produce a final volume of a 25 µL to perform the PCR using a T100™ thermal cycler (Bio-Rad, Singapore).

**Table 1:** Primer pairs and PCR conditions used for amplification of selected Aflatoxin PCR gene primer pairs and their condition.

Primer pair	Target gene	Primer sequence (5′-3′)	PCR conditions						
			1	2	3	4	5	6	Expected amplicon size
Nor1F Nor 1R	*AflD*	ACCGCTACGCCGGCACTCTCGGCAC GTT GGCCGCCAGCTTCGACACTCCG	94 °C:10min	94 °C:1min	65 °C: 1min	72 °C:2min	33	72 °C: mins	400 pb
Ver-IF Ver-1R	*AflM*	GCCGCAGGCCGCGGAGAAAGTGGT GGGGATATACTCCCGCGACACAGCC	95 °C:4mins	95 °C:1min	58 °C:1min	72 °C:30sec	30	72 °C:10mins	600 bp
Omt-IF Omt-1R	*aflP*	GTGGACGGACCTAGTCCGACATCAC GTCGGCGCCACGCACTGGGTTGGGG	94 °C:5mins	94 °C:1min	75 °C:2mins	72 °C:2mins	33	72 °C:10mins	797 bp
AflR-IF AflR-1R	*aflR*	TATCTCCCCCCGGGCATCTCCCGG GTCGGCGCCACGCACTGGGTTGGGG	95 °C:4mins	94 °C: 1min	60 °C:1min	72 °C:30sec	30	72 °C:10mins	1000 bp

#### 2.9.1. Identification of changes in the *Aspergillus* isolates at different treatment conditions.

The PCR products were electrophoresed for approximately an hour (80 volts; 400 A) in 1xTAE (Tris Acetate-EDTA) buffer on a 1.0% agarose gel containing 1.5 µL ethidium bromide. Gel was visualized using the gel documentation system (Bio-Rad Molecular Image® Gel DocTM XR+ with Image LabTM software, USA). The amplicons of different sizes were recorded and sequenced at Inqaba Biotech in South Africa. Chromas software version 2.6.6 was used to view the amplicon sequences. Molecular Evolutionary Genetics Analysis (MEGA) vs. 8 [36] was used for the alignment of multiple genomic sequences by the Muscle algorithm and phylogenetic analysis was done using the maximum likelihood method.

### 2.10. RNA extraction

The Trizol procedure was used to extract RNA. Fungal spores were mixed with 1 mL of Trizol for 10 min at 25 Hz in a TissueLyser II (Qiagen) machine. It was followed by adding 200 µL of chloroform and vortexing each sample for 15 sec. The reaction was centrifuged at 184000 x g for 20 min at 4^o^C, and the supernatant was then transferred to new tubes. Isopropanol (500 µL) was added, and the reaction was centrifuged once more for 20 min at 4 °C. The obtained pellet was cleaned or rinsed twice with 75% ethanol using a centrifuge at 12000 x g, at 4^o^C for 10min, then allowed to dry before adding RNAse water. RNA integrity and quantity were analysed using 1% agarose gel electrophoresis and Nanodrop 2000 (Thermo Scientific, Wilmington, DE, USA) Spectrophotometry, respectively.

### 2.11. Quantitative real-time PCR

RNA was reverse transcribed into complementary DNA (cDNA) using QuantiTect Reverse Transcription kit (Qiagen, Hilden, Germany), according to the manufacturer’s instructions. Gene expression was measured by StepOnePlus Real-Time PCR system (Applied Biosystems; Thermo Scientific™, Massachusetts, United States of America) using the fluorescent TaqMan Fast Advanced Master mix (Thermo Scientific TM, MA, USA). qPCR was performed using 4 µL of cDNA with 6 µL TaqMan Master Mix and TaqMan gene expression assays and qPCR conditions were 95 °C for 10 min, followed by 40 cycles of 95 °C for 15 s and 60 °C for 1 min. Expression of aflatoxin genes (*aflD, aflM, aflP*, and *aflR*) were investigated. RNA was normalised with β-tubulin. Data were analysed using 2 delta Ct ABI software.

### 2.12. Statistical analysis

Data were expressed as the mean ± SEM of three independent experiments. All statistical analyses were performed using GraphPad Prism 8 software (GraphPad Prism Software Inc. La Jolla, CA, USA). Means were separated using Tukey’s least significant difference at 5% level of significance.

## 3. Results

### 3.1. Phytochemical analysis of *U. epigea* bulb

In a qualitative analysis of the *U. epigea* bulb, four phytochemical tests for alkaloids, flavonoids, tannins, and saponins tested positive, and terpenoids tested negative as shown in Table 2. Phytochemicals play a significant role in reducing or capping agents during nanoparticle synthesis. They also help in improving the particle size [37] and providing the first line of defense against microorganisms [38].

**Table 2:** Qualitative analyses of *U. epigea* bulb phytochemicals.

Compounds	Phytochemical analysis
Alkaloids	+
Flavonoids	++
Saponins	+
Terpenoids	–
Tannins	++

+: presence and - absence

### 3.2. Inhibitory effect of *U. epigea* and Ag/ZnO nanoparticles on fungal growth

**Fig 1:**
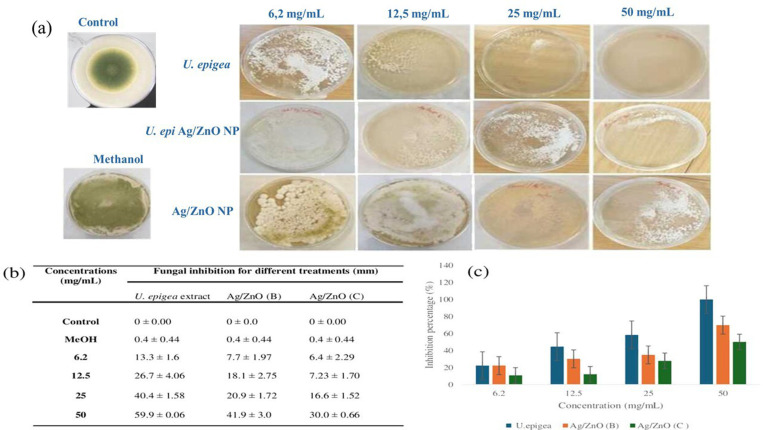
(a) Mycelial diameter (mm) determination on PDA plates, (b) Inhibition diameters, and (c) inhibition percentages of *Aspergillus flavus* treated with *U. epigea* extract and Ag/ZnO nanoparticles (Biosynthesized (B) and Conventional (C) at various concentrations. Each data point represents the mean ± Standard deviation (n = 3, per condition).

All tested concentrations reduced the mycelial growth of *Aspergillu*s fungus when compared to the control. Inhibition was observed in a dose dependant manner, low concentration (6.2 mg/mL) showed less inhibition (>20%), while high concentrations (50 mg/mL) showed a high level of inhibition of about 100% (Fig 1). Fig 1b and c summarizes the antifungal study conducted by measuring zones of inhibition against the tested fungi. The mycelial growth of fungal pathogens was significantly inhibited by incorporating various concentrations of treatments into PDA. A significant decrease in the growth of the mycelial colonies was observed in the treated group compared to the control. The diameters of *A. flavus* appeared much smaller at all concentrations than the control (Fig 1b). However, the positive control (vehicle-methanol) in Fig 1a shows changes in the growth and morphology concentration of the A*spergillus* and the change in the mycelia colour turning yellow green.

### 3.3. Effect of *U. epigea* on aflatoxin production

**Fig 2:**
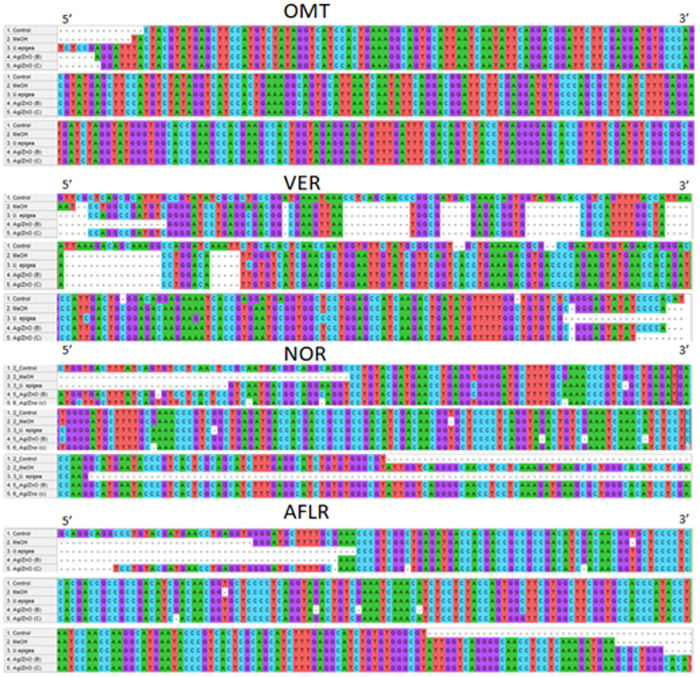
Nucleotide sequence alignment of aflatoxin genes *Nor, Omt*, *Ver* and *aflR* treated with *U. epigea* extract, Ag/ZnO (Biosynthesized and Conventional) nanoparticles from *A. flavus* with genomic DNA.

Fig 2 shows the effect of the *U. epigea* and Ag/ZnO nanoparticles on aflatoxin gene sequences. It is observed that treatment caused some mutations to the genes responsible for aflatoxin biosynthesis *No*r, *Omt*, *Ver*, and *AflR*. *U. epigea* showed fewer similarities for all the genes more especially *Ver* as it is a precursor of the AFB_1_ biosynthetic pathway of aflatoxin. Thus, can be assumed that aflatoxin synthesis was affected by the presence of the compounds in the *U. epigea.*

**Fig 3:**
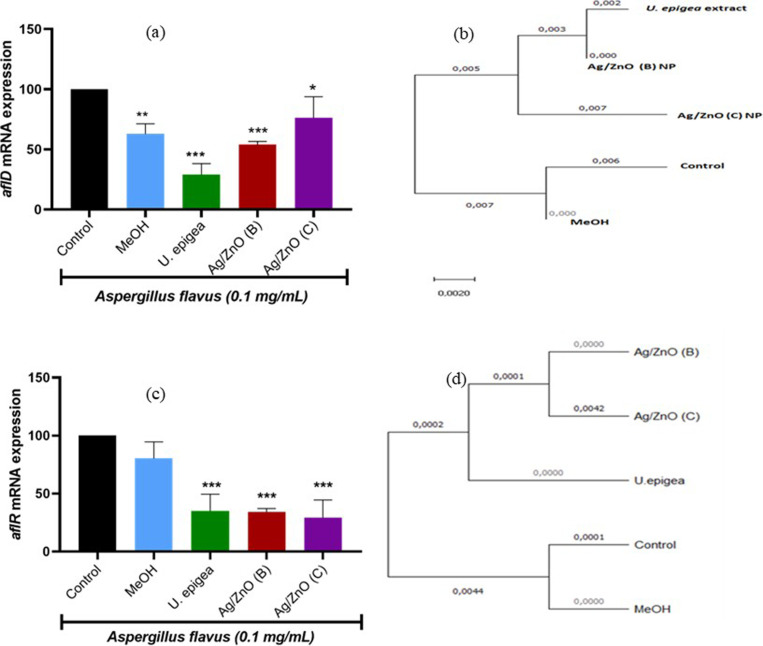
*Aspergillus flavus* mRNA expression (a) *aflD* and (c) *aflR* and phylogenetic analysis of (b) *aflD* and (d) *aflR* after exposure to 50 mg/mL of *U. epigea* extract and Ag/ZnO nanoparticles normalised to **β-tubulin.** Results are expressed as means ± SD of **n** = 5. The bars depict statistical differences at *P < 0.05, **P < 0.01, ***P < 0.001. MeOH = Methanol; Ag/ZnO (B) = biosynthesized Ag/ZnO; Ag/ZnO = conventional Ag/ZnO nanoparticles.

The effect of *U. epigea* extract and Ag/ZnO nanoparticles (biosynthesized and conventional) on the expression of aflatoxin synthesis genes in *A. flavus* is presented in Fig 3. It has been reported that *aflD* and *aflR* are two important key regulatory and structural genes, respectively involved in the biosynthetic pathway or production of aflatoxin. Real-time PCR results revealed that the expression of these genes was significantly downregulated (*P* < 0.005) by the treatments, with *U. epigea* having 50fold decrease when compared to Ag/ZnO nanoparticles. Compared to the controls, Ag/ZnO nanoparticles down-regulated the expression of *aflD* and *aflR* in *A. flavus* by more than 30-fold (Fig 3a and c). In the presence of *U. epigea,* Ag/ZnO (B), and Ag/ZnO (C), the relative expression of *aflD* was downregulated by 35%, 58%, and 72%, respectively, while *aflR* was downregulated by 42%, 40%, and 38%.

## 4. Discussion

The use of natural compounds for the development of eco-friendly pesticides has gained interest because of the hazardous influence of chemical ones on humans and the environment. *U. epigea* has antifungal properties which have led to its use in medicine for irritation and born breakages. Herein, *U. epigea* inhibited the *A. flavus* growth at all tested concentrations. This is in line with the findings obtained by Suleman [39], who stated a significant difference between mycelial growth values recorded on different concentrations of plant extract, and Ozbek *et al*. [40] found that growth inhibition levels increase with an increased concentration of antimicrobials used. At 50 mg/mL of *U epigea, A. flavus* production was completely inhibited (no *Aspergillus* growth), which could be related to the presence of bioactive chemicals that give fungus susceptibility [40]. Plant extract’s antimicrobial properties may cause damage to lipids, proteins, cell walls, cell membranes, and cellular organelles [41]. In addition, some *Thymus and Satureja* species have been shown to limit fungal growth and aflatoxin production [42]. It should also be noted from the literature that *U. epigea* has some alkaloids, phenolic, and flavonoid compounds and these compounds are known to possess antifungal properties [43,44]. Thus, the inhibition of *Aspergillus* can be due to the presence of these compounds. In addition to this, the tannins from medicinal plants possess remarkable toxic activity against fungi and they may assume pharmacological importance [45]. Furthermore, saponins are a special class of glycosides that have soapy characteristics and it is considered active antifungal agents [46].

However, the negative control (vehicle-methanol) in Fig 1 shows some changes in the morphology of the A*spergillus*, the mycelia were turning yellow-green, this may be due to some chemical reaction. Due to the solubility of antimicrobial active components in methanol, an organic solvent used for extraction [41] growth inhibition that appears as clear on extract-treated plates is produced since the active compounds are more effective in polar solvents than in water. The aqueous extract experiment was carried out, but the results were not reported because there was no inhibition which was likely caused by the fact that the active phytocompounds were not absorbed in a nonpolar solvent.

The inhibition percentage of the biosynthesized Ag/ZnO was more than the conventional Ag/ZnO, which could be attributed to the presence of phytochemicals and the size of the nanoparticles in the biosynthesized Ag/ZnO. Different nanomaterials can kill fungal cells, and the formation of pores in the membrane causes the release of biomolecules from cells, resulting in cell death. According to research on the inhibition effect of nanoparticles on the fungus *Aspergillus*, the cell membrane is the target site for nanoparticles. The antimicrobial activity of Ag, which is thought to interact with DNA replication, may also contribute to the nanoparticle treatment’s inhibition range [47], and can liberate Ag+ ions and bind to proteins with cysteine moieties on the plasma membrane and can cause damages physiologically or biologically resulting in compromised membrane stability. Consequently, nanoparticles due to their large surface area penetrate the cytoplasm and inactivate protein enzyme functions thus lead to cell death [48].

In light of the findings of Borges *et al.* [49], the growth of the fungus and the inhibition of AFB_1_ production may be understood. According to the authors, plant extracts function as antioxidants to inhibit aflatoxins by squelching free radicals, preventing their reproduction, and turning them into less harmful molecules. However, the morphological identification could not determine if the aflatoxin production was inhibited hence the biochemical analysis of aflatoxin-producing genes was determined.

The RT-PCR results confirmed the downregulation of the aflatoxin-producing genes, which indicates that *U. epigea* has higher antifungal and anti-aflatoxigenic activity against *Aspergillus flavus*. The downregulation of the regulatory gene *aflR* could have subsequently suppressed the expression of the structural genes *aflD*, as it is known that *aflD* encodes a reductase involved in the conversion of the first stable aflatoxin biosynthesis intermediate, (*Nor* to averantin), thus its downregulation expression could have inhibited aflatoxin production in the early stages of the biosynthetic pathway *aflM* and *aflP*. The *aflM* (VER) gene was not expressed in the RT-PCR, which may have been caused by a gene mutation brought on by treatment exposure. Fig 3 shows that when compared to the control, there were some deletions and gaps in the majority of the nucleotides. Particularly in toxigenic *Aspergillus* species, the *aflP* (omt-A) gene in *Aspergillus* strains is essential for aflatoxin synthesis [50]. The formation of aflatoxin depends on the conversion of sterigmatocystin to o-methylsterigmatocystin, which is carried out by the omt-A gene [51]. Due to the fact that *aflD* is responsible for measuring the amount of AFB_1_ produced by *Aspergillus* strains, the downregulation of *aflD* expression may imply that less AFB_1_ was produced following the treatment. As *aflR* encodes for a transcription factor of the Zn2Cys6-type [52], which is required to transcribe the aflatoxin gene cluster, the results of this study showed that *aflR* was more downregulated by *U. epigea* and Ag/ZnO nanoparticles. This could be a significant indicator of the strain’s inability to produce aflatoxin. It was also observed in the phylogeny that the treatments had more effect on the *Aspergillus*, as shown in Fig 3b and d that *U. epigea* was not close to the control and a huge difference was observed on the conventional Ag/ZnO. Furthermore, some effects of the treatment might have impeded the crucial enzymatic mechanism that produced sterigmatocystin [53]. The treatment might have caused mutations in the aflatoxin biosynthetic pathway due to a distortion in the order of enzyme production leading to aflatoxin biosynthesis. Many transcription factors, including pH, carbon, or nitrogen sources, have the power to influence chromatin structure by promoting or suppressing gene expression. These elements might encourage the adherence or mismatching of sequence-specific primers to locations in the target genes’ promoter regions, resulting in complexes that help a different transcription process [50]. Another factor that could affect the expression of aﬂatoxin biosynthesis genes is the location of the chromosome in the genome of presumptive toxigenic fungi [54].

Moreover, compounds that inhibit mycotoxin production can act by altering the environmental and physiological modulators of mycotoxin biosynthesis or by altering the signal transduction pathways upstream of the biosynthetic pathway. Several studies have already shown that some of these natural products can inhibit AFB_1_ production via transcriptional down-regulation of the genes involved in AFB_1_ synthesis [55,56]. In this study, the antifungal effect of *U. epigea* could be attributed to the reaction of bufadienolide with reactive groups of fungal enzymes, possibly via reactions with sulfhydryl groups or nonspecific interactions with proteins [57]. The molecular mechanism of action suggests that phytochemicals present in *U. epigea* bound the *A. flavus* phospholipid cell membrane resulting in increased permeability and decreased fluidity [58] and also reduced ergosterol content, and increased malondialdehyde level [59]. Additionally, they can affect fungal cell wall integrity, leading to the leakage of alkaline phosphatase, alterations in the contents of β-1,3-glucan and chitin, and collapse of spores that result in a cell wall autolysis-like phenotype [60].

## 5. Conclusions

*Urginea epigea* inhibited the growth of *A. flavus* thus aflatoxin production was reduced. A significant inhibition percentage of mycelial growth, higher than 70%, was obtained with the four concentrations of treatments. Aflatoxin production was significantly reduced with the presence of the treatment although, in some cases, at low concentrations, they stimulated mycotoxin production. Therefore, controlling the dose of natural fungicides is crucial since suboptimal concentrations could lead to stimulation of both growth and toxin accumulation. This reduction in growth suggests that phytochemical compounds could be used alone or in conjunction with other substances or processes to control the presence of toxic metabolites in feed. In addition, *U. epigea* down-regulated critical AF synthesis genes (*aflD* and *aflR*) in *A. flavus*. The *U. epigea* extract must be subjected to further study to characterize other compounds, define toxicity, and evaluate economic feasibility. More importantly, it can be employed as an alternative antifungal agent for fungi. The present study provides a scientific basis for further study of the inhibiting mechanisms. In any case, *U. epigea* bulb may provide an alternative strategy to the use of synthetic chemical fungicides to ensure food safety, which deserves additional studies to assess more details regarding their practical applications. Our future studies will use antifungal standards are positive controls.
